# Checklist of the fly families Chyromyidae and Heleomyzidae (Diptera) of Finland

**DOI:** 10.3897/zookeys.441.7507

**Published:** 2014-09-19

**Authors:** Jere Kahanpää

**Affiliations:** 1 Finnish Museum of Natural History, Zoology Unit, P.O. Box 17, FI-00014 University of Helsinki, Finland

**Keywords:** Species list, Finland, Diptera, biodiversity, faunistics

## Abstract

A Finnish checklist of the sphaeroceroid fly families Chyromyidae and Heleomyzidae is provided.

## Introduction

The superfamily Sphaeroceroidea is a medium-sized one, with two families of moderate diversity, Sphaeroceridae (1550 species) and Heleomyzidae (~720 species), and the small family Chyromyidae. The enigmatic afrotropical *Mormotomyia hirsuta* Austen, 1936 was once placed near Sphaeroceridae but it is now seen as an ephydroid fly (Kirk-Spriggs et al. 2011). McAlpine (2007) has proposed an alternative concept for Sphaeroceroidea with Sphaeroceridae and Heleomyzidae united as a single family called Heteromyzidae. This proposal has not gained significant support and for the purposes of this checklist the traditional concept of family Sphaeroceridae is retained.

There is no general agreement on the relationships of various heleomyzid tribes. Several different schemes for subfamilies have been proposed (see McAlpine 2007, McAlpine and Woodley 2010). Some taxa treated here as heleomyzids (primarily Trixoscelidinae, Chiropteromyzinae, Heteromyzinae and Borboropsini) may deserve full family status. As a conservative approach this checklist follows Marshall (2012) and keeps them as subfamilies and tribes. The heleomyzid subfamilies and tribes are listed alphabetically.

The Finnish chyromyids are small yellow flies with (at least while alive) iridescent blue or green eyes. Chyromyids are rarely collected and little is known about their ecology or the proper place of the family within Sphaeroceroidea. They may actually be a specialized lineage arising from within Heleomyzidae *sensu lato*.

Two of the three sphaeroceroid families are treated in this paper. The largest, Sphaeroceridae, is covered in a separate paper in this issue of ZooKeys. The Finnish species of Heleomyzidae and Chyromyidae were last listed by Hackman (1980).

**Table 1.** Number of species by family.

Family	Number of species in			Level of knowledge
	World (Pape et al. 2011)	Europe	Finland	
Chyromyidae	138	59	4	poor–average
Heleomyzidae	727	175	61	average

## Checklist

suborder Brachycera Macquart, 1834

clade Eremoneura Lameere, 1906

clade Cyclorrhapha Brauer, 1863

infraorder Schizophora Becher, 1882

clade Muscaria Enderlein, 1936

parvorder Acalyptratae Macquart, 1835

superfamily Sphaeroceroidea Macquart, 1835

**CHYROMYIDAE** Hendel, 1916

CHYROMYINAE Hendel, 1916

***CHYROMYA*** Robineau-Desvoidy, 1830

*Chyromya flava* (Linnaeus, 1758)

*Chyromya oppidana* (Scopoli, 1763)

***GYMNOCHIROMYIA*** Hendel, 1933

*Gymnochiromyia flavella* (Zetterstedt, 1848)

= *minima* (Becker, 1904)

*Gymnochiromyia inermis* (Collin, 1933)

**HELEOMYZIDAE** Westwood, 1840

BORBOROPSINAE Griffiths, 1972

***BORBOROPSIS*** Czerny, 1902

*Borboropsis puberula* (Zetterstedt, 1838)

= *fulviceps* (Strobl, 1898)

CHIROPTEROMYZINAE Frey, 1952

***CHIROPTEROMYZA*** Frey, 1952

*Chiropteromyza broersei* (de Meijere, 1946)

= *wegelii* Frey, 1952

***NEOSSOS*** Malloch, 1927

= ***Ornitholeria*** Frey, 1930

*Neossos nidicola* (Frey, 1930)

HETEROMYZINAE Fallén, 1820

***HETEROMYZA*** Fallén, 1820

*Heteromyza atricornis* Meigen, 1830

*Heteromyza oculata* Fallén, 1820

*Heteromyza rotundicornis* (Zetterstedt, 1846)

***TEPHROCHLAMYS*** Loew, 1862

*Tephrochlamys flavipes* (Zetterstedt, 1838)

*Tephrochlamys rufiventris* (Meigen, 1830)

= *lapponica* (Czerny, 1924)

*Tephrochlamys steniusi* Frey, 1930

*Tephrochlamys tarsalis* (Zetterstedt, 1847)

HELEOMYZINAE Westwood, 1840

tribe Heleomyzini Westwood, 1840

***GYMNOMUS*** Loew, 1863

*Gymnomus amplicornis* (Czerny, 1924)

***HELEOMYZA*** Fallén, 1810

= ***Helomyza*** Fallén, 1820 emend.

= ***Leria*** Robineau-Desvoidy, 1830

**sg. *Heleomyza*** Fallén, 1810

*Heleomyza borealis* Boheman, 1865

= *czernyi* Collart, 1933

= *modesta* misid.

*Heleomyza hackmani* Frey, 1950

*Heleomyza pleuralis* (Becker, 1907)

*Heleomyza serrata* (Linnaeus, 1758)

***MORPHOLERIA*** Garrett, 1921

**sg. *Spanoparea*** Czerny, 1924

*Morpholeria dudai* (Czerny, 1924)

*Morpholeria kerteszii* Czerny, 1924

*Morpholeria obscuriventris* (Zetterstedt, 1847)

*Morpholeria ruficornis* (Meigen, 1830)

***NEOLERIA*** Malloch, 1919

*Neoleria inscripta* (Meigen, 1830)

= *minuta* (Zetterstedt, 1838)

*Neoleria prominens* (Becker, 1897)

= *tibialis* misid.

*Neoleria ruficauda* (Zetterstedt, 1847)

*Neoleria ruficeps* (Zetterstedt, 1838)

***SCOLIOCENTRA*** Loew, 1862

**sg. *Chaetomus*** Czerny, 1924

*Scoliocentra confusa* (Wahlgren, 1918)

*Scoliocentra flavotestacea* (Zetterstedt, 1838)

**sg. *Leriola*** Gorodkov, 1962

*Scoliocentra brachypterna* (Loew, 1873)

*Scoliocentra nigrinervis* (Wahlgren, 1918)

**sg. *Scoliocentra*** Loew, 1862

*Scoliocentra dupliciseta* (Strobl, 1894)

*Scoliocentra scutellaris* (Zetterstedt, 1838)

*Scoliocentra villosa* (Meigen, 1830)

tribe Oecotheini Gorodkov, 1972

***ECCOPTOMERA*** Loew, 1862

*Eccoptomera infuscata* Wahlgren, 1918

*Eccoptomera longiseta* (Meigen, 1830)

*Eccoptomera marginicornis* Czerny, 1924

*Eccoptomera microps* (Meigen, 1830)

*Eccoptomera obscura* (Meigen, 1830)

*Eccoptomera ornata* Loew, 1862

*Eccoptomera pallescens* (Meigen, 1830)

***OECOTHEA*** Haliday, 1837

*Oecothea fenestralis* (Fallén, 1820)

tribe Orbelliini Gorodkov, 1972

***ORBELLIA*** Robineau-Desvoidy, 1830

*Orbellia nivicola* Frey, 1913

SUILLIINAE Wahlgren, 1917

***SUILLIA*** Robineau-Desvoidy, 1830

= ***Allophyla*** Loew, 1862

*Suillia affinis* (Meigen, 1830)

*Suillia apicalis* (Loew, 1862)

*Suillia atricornis* (Meigen, 1830)

*Suillia bicolor* (Zetterstedt, 1838)

*Suillia femoralis* (Loew, 1862)

*Suillia flava* (Meigen, 1830)

*Suillia flavifrons* (Zetterstedt, 1838)

= *nudipes* (Czerny, 1932)

*Suillia fuscicornis* (Zetterstedt, 1847)

*Suillia humilis* (Meigen, 1830)

= *inornata* (Loew, 1862)

*Suillia laevifrons* (Loew, 1862)

*Suillia lineitergum* (Pandellé, 1901)

= *stroblii* (Czerny, 1904)

*Suillia lurida* (Meigen, 1830)

*Suillia mikii* (Pokorny, 1886)

*Suillia nemorum* (Meigen, 1830)

*Suillia pallida* (Fallén, 1820)

*Suillia parva* (Loew, 1862)

= *collini* Hackman, 1972

= *flavifrons* auct. nec (Zetterstedt, 1838)

*Suillia quadrilineata* Czerny, 1924

*Suillia vaginata* (Loew, 1862)

TRIXOSCELIDINAE Hendel, 1916

***TRIXOSCELIS*** Rondani, 1856

*Trixoscelis frontalis* (Fallén, 1823)

? = *canescens* misid. (see Notes)

*Trixoscelis marginella* (Fallén, 1823)

*Trixoscelis obscurella* (Fallén, 1823)

*Trixoscelis similis* Hackman, 1970

## Notes

***Chyromya oppidana* (Scopoli, 1763)**. Found only inside houses and farm buildings in Finland.

***Orbellia nivicola* Frey, 1913**. This species was synonymized with *Orbellia myiopiformis* R.-D. by Storå (1958), but Frey (1958) defended its validity. The status of *Orbellia nivicola* as a species needs verification.

***Trixoscelis canescens* (Loew, 1865)**. This species was originally described on the basis of a single female. Soós (1979) examined the type and revived the name from synonymy with *Trixoscelis frontalis*. Woźnica (2008) provided an illustrated diagnosis for *Trixoscelis canescens* and synonymized *Trixoscelis gigans* Carles-Tolrá, 2001 and *Trixoscelis fumipennis* Papp, 2005 with it. The species was recently recorded from Finland by Flinck and Kahanpää (2013). Specimens with darkened costal veins and dorsal abdominal surfaces, both proposed diagnostic characters of *Trixoscelis canescens*, are common among Finnish *Trixoscelis frontalis* material (see Fig. 8 in Flinck and Kahanpää 2013). Finnish males with these features have genitalia identical with those illustrated for *Trixoscelis frontalis* by Hackman (1970) and quite unlike the genitalia of *Trixoscelis gigans* (= *fumipennis* Papp). The male specimen mentioned in Flinck and Kahanpää (2013) was later dissected and it belongs to *Trixoscelis frontalis*. The external characters (darkened costa and dorsum of abdomen) can not be used to reliably separate *Trixoscelis canescens* from *Trixoscelis frontalis*. The Finnish records of *Trixoscelis canescens* are probably all misidentifications of *Trixoscelis frontalis*.
